# La medicina che non c’è

**DOI:** 10.1007/s40619-022-01024-5

**Published:** 2022-01-21

**Authors:** Silvia Grottoli

**Affiliations:** Dipartimento di Scienze Mediche, Università degli Studi di Torino, Torino, Italia

Commento a:

**La medicina che non c’è.**

**O. Davini.**

**(2021) Edizioni Dedalo, Bari, p. 96**

“Non si elimina l’incertezza, si negozia con essa”, scriveva Edgar Morin nel 2015. La pandemia ha reso evidente la nostra difficoltà di comprendere la scienza e la sua complessità e il caos comunicativo ha evocato una medicina che non c’è. Questo libro è stato scritto con l’intento di imparare a convivere con le inevitabili incertezze e non essere in balia degli istinti o della peggiore politica. Per credere nella vera medicina e non nelle illusioni.

Questa è la breve sintesi di questo testo scritto da un medico per tutti e quindi anche per noi medici.

Partendo dalla pandemia da Sars-CoV-2, Davini mette l’accento su una serie di contraddizioni con cui viene vista la Medicina moderna e che ha generato una sorta di cortocircuito tra la scienza medica e la popolazione. Infatti, per alcuni la Medicina può risolvere ogni nostro problema di salute, mentre per altri la scienza è inutile per fronteggiare le sfide future.

L’autore ha analizzato le molte domande e i molti dubbi che si pongono i comuni cittadini e ha cercato di dare una risposta, almeno parziale. Il libro suggerisce alcuni spunti di riflessione a cui ognuno di noi, medici, non si può sottrarre per meglio comprendere questo malessere che percorre l’Italia negli ultimi tempi.

**Figure 1:**
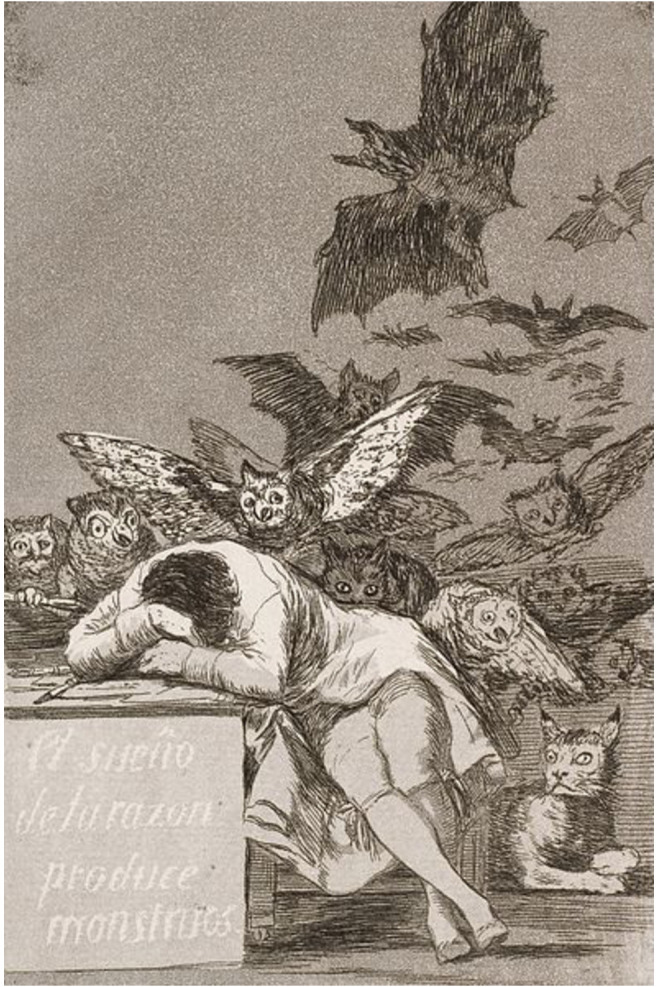

Bisogna che facciamo i conti con la medicina delle incertezze perché la medicina in sé apre molte porte e risponde a molte domande ma ad ogni domanda risposta, ne nasce un’altra. Perché la sete di conoscenza non finisce mai: una certezza di oggi potrebbe rivelarsi falsa verità in futuro e la scienza è sempre più vicina alla verità che, però, non si è mai certi di raggiungere. Fortunatamente, accorre in nostro aiuto il metodo scientifico, che rende la scienza democratica ma rigorosa.

Davini affronta il problema della complessità e della sua differenza rispetto al “complicato/difficile”. Il complicato può essere affrontato e risolto con le competenze che, a volte, non sono sufficienti per affrontare un problema complesso. La complessità esprime l’interconnessione dei sistemi, la quale permette di far emergere proprietà non possedute dai singoli ma solo dalla loro interazione. A questo argomento si collega il concetto dell’intelligenza cumulativa di gruppo.

Davini accenna e ci suggerisce di approfondire il concetto di *One Health*, ossia l’interdipendenza tra la salute del pianeta e di chi lo abita.

Infine, il libro affronta la medicina delle illusioni e dell’ignoranza (*La medicina che non c’è*). L’ignoranza che, in passato, le classi dominanti hanno sempre usato per controllare il popolo e che oggi viene sventolata proprio da coloro che vorrebbero emanciparsi. Ove c’è ignoranza, l’irrazionale prevarrà sempre sul razionale e l’esempio emblematico è quello dei vaccini. Analizzando il concetto di rischio, degli effetti collaterali delle terapie e della sovradiagnosi, l’autore approfondisce il “mito” molto diffuso, ma ovviamente falso, che non si possa più morire. Perché la scienza e la tecnologia di cui disponiamo ci impediranno di soccombere. Da questa credenza, chiaramente smentita negli ultimi due anni, deriva il grande scettiscismo a cui abbiamo e a cui stiamo ancora assistendo nel nostro paese e la conseguente sfiducia nella Medicina e nella Scienza (Fig. 1).

Il libro è molto più di questo, e vale davvero la pena di leggerlo.

Forse una volta sola non basta ma è un piccolo libro di 90 pagine, con abbondante bibliografia, molto ben scritto e non pesante. Forse leggerlo due volte non ci costerà fatica.

